# Regulatory Role of Fc Receptor in mIgM^+^ B Lymphocyte Phagocytosis in Flounder (*Paralichthys olivaceus*)

**DOI:** 10.3389/fimmu.2021.804244

**Published:** 2021-12-17

**Authors:** Yanbo Hao, Xiaoqian Tang, Jing Xing, Xiuzhen Sheng, Heng Chi, Wenbin Zhan

**Affiliations:** ^1^ Laboratory of Pathology and Immunology of Aquatic Animals, KLMME, Ocean University of China, Qingdao, China; ^2^ Laboratory for Marine Fisheries Science and Food Production Processes, Qingdao National Laboratory for Marine Science and Technology, Qingdao, China

**Keywords:** *Paralichthys olivaceus*, Fc receptor, phagocytosis, mIgM^+^ B lymphocytes, opsonization

## Abstract

Fc receptor (FcR) is an important opsonin receptor on the surface of immune cells, playing an important role in antibody-dependent cell-mediated immunity. Our previous work found that the FcR of flounder showed a marked expression response in phagocytizing IgM^+^ B cell, which suggested that FcR might participate in regulating Ig-opsonized phagocytosis. In this paper, in order to elucidate the potential role of FcR in mediating phagocytosis of IgM^+^ B cell, flounder anti-*E. tarda* serum was prepared and complement-inactivated for the use of *E. tarda* opsonization, and the sera of healthy flounder were used as control. Flow cytometric analysis showed that the phagocytosis rates of antiserum-opsonized *E. tarda* in peripheral blood mIgM^+^ B lymphocytes were significantly higher than the control group, and higher phagocytosis rates of mIgM^+^ B lymphocyte could be detected with an increasing incubation time ranging from 1 to 5 h. The phagocytosis rates of antiserum-opsonized *E. tarda* by mIgM^+^ B lymphocyte for an incubation time of 1, 3 or 5 h were 51.1, 63.0, and 77.5% respectively, which were significantly higher than the phagocytosis rates in the control groups with 40.2, 50.9, and 63.8%, respectively. While the Fc fragment of IgM on the surface of opsonized *E. tarda* was blocked by rabbit anti-flounder IgM polyclonal antibodies, phagocytosis rates of mIgM^+^ B lymphocyte decreased significantly compared with the unblocked group. Moreover, the proportion of mIgM^+^ B lymphocytes with higher intracellular reactive oxygen species (ROS) levels rose to 32.1% from the control level of 23.0% after phagocytosis of antiserum-opsonized *E. tarda*. FcγRII and Syk were found to be significantly upregulated, while FcγRIII was significantly downregulated in the mIgM^+^ B lymphocytes post phagocytosis. Furthermore, when FcγRII of mIgM^+^ B lymphocytes was blocked by the prepared antibodies, their phagocytosis rate of antiserum-opsonized *E. tarda* was 39.0%, which was significantly lower than the unblocked group of 54.0%. These results demonstrate that FcR plays a critical role in mediating phagocytosis and bactericidal activity of mIgM^+^ B lymphocytes, which would facilitate an improved understanding of the regulatory roles of FcR in phagocytosis of teleost B lymphocytes.

## Introduction

The function of phagocytosis refers to the recognition and ingestion of large particles into phagosomes and it plays a significant role in the elimination of microbial pathogen and apoptotic cells (1). The phenomenon of phagocytosis primarily exists in macrophages, monocytes, and neutrophils (2). But recent studies have shown that B cells also possess the abilities of phagocytosis. In mammals, mice B1 cells have been proved to have the abilities of phagocytosis (3). In amphibians, for example, *Xenopus laevis*, B cells have the potential phagocytic abilities (4). The teleost’s IgM^+^ B lymphocytes were also proved to have phagocytic activities toward outside particles in different species, such as sea bass (*Lateolabrax japonicus*) (5), half-smooth tongue sole (*Cynoglossus semilaevis*) (6), flounder (*Paralichthys olivaceus*) (7), lumpfish (*Cyclopterus lumpus*) (8) and Nile tilapia (*Oreochromis niloticus*) (9).

The Fc receptor (FcR) is mostly functional as a complex of multiple subunits and gives play to significant functions, exactly in antibody-dependent cellular phagocytosis (ADCP) and antibody-dependent cell-mediated cytotoxicity (ADCC) (10, 11). FcRs are present in mammals (12), birds (13), amphibians (14), fish (15–18), and other vertebrates and are mainly expressed on the surface of leukocytes, such as B cells, T cells, and natural killer (NK) cells. In higher vertebrates, while FcγRII recognizes Fc fragment, Src family kinases induce the phosphorylation of spleen tyrosine kinase (Syk) and ITAM motif, thereby activating the downstream phagocytosis-related genes (19–21). Recent studies have shown that FcγRII was expressed on multiple types of immune cells and are divided into three types: FcγRIIa, FcγRIIb, and FcγRIIc, of which only FcγRIIb was expressed on B cells in mammals (22). In addition, FcγRIII was divided into two types, FcγRIIIa and FcγRIIIb, and played important roles in mammal ADCC (23). Among them, FcγRIIIa was a transmembrane protein, mainly found in monocytes and macrophages, and was essential for anti-inflammatory response (24). FcγRIIIb is a non-transmembrane protein, which attaches to the membrane surface of immune cells at resting state and sheds from the membrane when binding to the IgG Fc fragment, resulting in a significant decrease in the expression levels of FcγRIII in nature killer (NK) cells (25). In teleosts, previous studies have only shown that IgM-opsonized *Aeromonas hydrophila* could significantly increase the phagocytosis rates of rainbow trout IgM^+^ B lymphocytes (4), but the role of FcR in this immunological process is still unknown. Therefore, in order to reveal the function of FcR in teleost mIgM^+^ B lymphocytes ADCP, we selected the flounder as the experimental animal and further explored the roles of FcγRII and FcγRIII in this process.

In this study, in order to illustrate the role of FcR in the ADCP process, the phagocytosis capability of flounder peripheral blood mIgM^+^ B lymphocytes were examined while *Edwardsiella tarda* bacteria were serum opsonized and also Fc or FcγRII was blocked. Furthermore, the expressions of FcγRII, FcγRIII, and Syk were detected in magnetic bead-sorted phagocytosed mIgM^+^ B lymphocytes, thus elucidating the signaling events that trigger ADCP. This research will broaden our understanding of the FcR functions in B cell phagocytosis and provide further insight into the role of B cells in teleosts’ innate immunity.

## Material and Methods

### Ethics Statement

This study was brought into force in severe accordance with the ethical criterion and the objective of the “Regulations for the Administration of Affairs Concerning Experimental Animals” proclaimed by the State Science and Technology Commission of Shandong Province. This study was also allowed by the Committee of the Ethics on Animal Care and Experiments at the Ocean University of China. The flounders were anesthetized with ethyl 3-amino-benzoate-methanesulfonic acid (MS222) before killing prior the experiment.

### Fish

Flounders (800 ± 40 g) were purchased from an aquafarm in Rizhao, Shandong Province, PR China. The fishes were communally reared in a pond including oxygen-rich and filtered seawater at 20.0 ± 1.0°C for seven days before the experiment.

### Preparation of Flounder Antiserum and Rabbit Anti-IgM Polyclonal Antibodies

The pathogenic *E. tarda* isolated from diseased flounder were cultured and inactivated by formalin as described before (26). Next the inactivated bacteria were adjusted to 1.0 × 10^8^ CFU/ml with 0.1 M phosphate buffered saline (PBS), and then mixed with Freund’s complete adjuvant (1:1, V/V). Each flounder was intraperitoneally injected with 200 μl emulsified *E. tarda*, and injected with the same volume of sterile PBS which was administrated as the negative control. After 2 weeks, the inactivated-*E. tarda* emulsified with Freund’s incomplete adjuvant (1:1, V/V) was given as a booster immunization. Blood samples were collected from the tail vein 35 days after the first immunization, and allowed to clot at room temperature for 1 h, then centrifuged at 4 °C at 8,000*g* for 15 min to obtain the anti-*E. tarda* serum.

In order to produce polyclonal antibodies against flounder IgM, the flounder IgM was purified from serum according to the procedure described previously with some modifications (27, 28). Briefly, the crude extract of IgM was isolated from the serum by salting out with 50% saturated ammonium sulfate and then purified using HiTrap^®^ Protein A column (GE Healthcare) by protein purification system (AKTA prime, Amersham). The purity of IgM was examined by SDS-PAGE, and its concentration was determined using the Bradford method and adjusted to 1.0 mg/ml using PBS. New Zealand white rabbits were then immunized with purified IgM to produce polyclonal antibodies according to previous procedure (29).

### Characterization of Flounder Antiserum and Rabbit Anti-IgM Polyclonal Antibodies

We first tested the characteristics of the prepared flounder anti-*E. tarda* serum. Briefly, wells of flat-bottom microplates (96-wells, Costar) were coated overnight with 100 μl/well of 1.0 × 10^8^ CFU/ml *E. tarda* at 4°C. The wells were washed three times with 0.1 M PBS, 0.1% Tween 20, and pH 7.4 (PBST) and then blocked with 3% BSA in PBS for 1 h at 37°C. After washing, the serum (1:100 diluted in PBS) sampled from vaccinated flounder was added 100 μl per well and incubated for 2 h at 28°C. Following washing, each well was added with 100 μl monoclonal antibody (mAb) 2D8 against flounder IgM (1:2,000 diluted in PBS), which was previously prepared by our lab (28). After 1 h incubation at 37°C, the goat-anti-mouse Ig-alkaline phosphatase conjugate (Sigma) diluted 1:5,000 in PBS was added and incubated for 45 min at 37°C. After the last washing, 100 μl 0.1% (w/v) p-nitrophenyl phosphate (pNPP, Sigma) in 50 mM carbonate-bicarbonate buffer (pH 9.8) containing 0.5 mM MgCl_2_ was used for color development for 30 min at 25°C before being measured by an automatic enzyme-linked immunosorbent assay (ELISA) reader (TECAN, Männedorf, Switzerland). For indirect fluorescence assay (IFA), the diluted *E. tarda* were settled onto glass slides for 2 h and fixed with 4% (V/V) paraformaldehyde for 20 min at 20°C, followed by the prepared antiserum which was added to glass slides and incubated for 2 h at 28°C; the healthy flounder sera were used as control. Then mAbs 2D8 (1:2,000) was added after three washings with PBST for 1 h at 37°C. Next the slides were incubated with Alexa Fluor^®^ 649 goat anti-mouse IgG (Invitrogen, Molecular Probes) for 45 min at 37°C. After the last washing, the slides were fixed with 90% glycerin before observation under a fluorescence microscope (Invitrogen™ EVOS™ FL Auto 2). Next, the prepared flounder anti-*E. tarda* serum was used for the opsonization of the *E. tarda*.

Meanwhile, the properties of the prepared rabbit anti-IgM polyclonal antibodies were also characterized. In brief, for western blotting, the flounder serum was separated by SDS-PAGE and transferred to PVDF membrane (Merck Millipore, Germany). The membrane was blocked with PBS containing 3% bovine serum albumin (BSA), and then incubated with the prepared rabbit anti-IgM polyclonal antibodies (diluted at 1:2,000 in PBS) at 37°C for 1 h and healthy rabbit sera were used as negative control. After washing thrice with PBST, goat-anti-rabbit IgG-alkaline phosphatase conjugate (Merck Millipore) was used to detect antibodies binding at 37°C for 1 h. After washing, the reactive bands were visualized for 5 min in the substrate solution (100 mM NaCl,100 mM Tris, 5 mM MgCl_2_, pH 9.5) containing NBT (Sigma) and BCIP (Sigma). The prepared rabbit anti-IgM polyclonal antibodies were used for blocking experiments later.

### Phagocytosis Assay

Complement in the prepared sera from healthy and vaccinated flounder were inactivated by water bath at 56°C for 30 min (30). The *E. tarda* was adjusted to the concentration of 1.0 × 10^10^ CFU/ml, and then incubated with complement-inactivated sera at a volume ratio of 1:10 for 5 h with gentle shaking at 28°C. After centrifugation at 10,000*g* for 5 min, the opsonized *E. tarda* was obtained and diluted with PBS to 1.0 × 10^8^ CFU/ml. Next, the healthy flounder sera and antiserum-opsonized *E. tarda* were labeled with fluorescein isothiocyanate (FITC) in preparation for subsequent phagocytosis experiments (5).

The leukocytes isolated by Percoll gradient from the three flounders were handled as three individual samples, which were then counted and diluted to 1.0 × 10^7^ cells/ml with L-15 medium. The 1,000 μl cell suspensions were incubated with antiserum-opsonized *E. tarda* for 1, 3 or 5 h at 22°C in 24-well culture plates, while the healthy flounder serum-opsonized *E. tarda* was used as negative control. The ratios of cells/bacteria were all set as 1:40. Next the non-adherent cells were collected and mixed with adherent cells loosened by the means of trypsin-EDTA. Non-ingested bacteria were removed by glucose cushion (3 ml PBS, pH 7.3, with 3% (W/V) BSA and 4.5% (W/V) D-glucose) and centrifuged at 100*g* for 10 min at 4°C. After ingestion, the isolated cells were suspended in 1,000 μl PBS containing 5% (V/V) newborn calf serum (NCS), followed by incubation with anti-IgM mAbs as primary antibody. Washing with PBS containing 5% (V/V) NCS, the cells were labeled with Alexa Fluor^®^ 649 goat anti-mouse IgG (Invitrogen, Molecular Probes). After the last washing, the phagocytosis rates of mIgM^+^ B lymphocyte were measured by a BD Accuri C6 Flow Cytometer (Becton Dickinson. USA). For determining cellular reactive oxygen species (ROS), mIgM^+^ B lymphocyte was stained by ROS Assay Kit (Beyotime) after being phagocytosed by antiserum-opsonized *E. tarda* for 2 h at 22°C, where healthy flounder serum-opsonized *E. tarda* was used as control.

In order to reversely illustrate the function of Fc fragment of flounder IgM in antibody-dependent cellular phagocytosis (ADCP), we used prepared rabbit anti-IgM antibodies to block the Fc fragment on the surface of antiserum-opsonized *E. tarda*. Briefly, the rabbit anti-IgM polyclonal antibodies (diluted in 1:1,000) were added to the flounder antiserum-opsonized *E. tarda* (1.0 × 10^10^ CFU/ml) then they reacted with each other at an incubator temperature of 37°C for 12 h, where the healthy flounder serum-opsonized *E. tarda* was used as negative control. Next the treated *E. tarda* was acquired by centrifugation at 10,000*g* for 5 min and adjusted to 1.0 × 10^8^ CFU/ml. The experimental and measuring methods of phagocytosis rates and production of cellular ROS in mIgM^+^ B lymphocyte were the same as the previous description. Finally, phagocytosis of *E. tarda* treated the mIgM^+^ B lymphocytes in different ways were observed by fluorescence microscopy (Olympus DP70, Tokyo, Japan) as described above.

### Detection of ADCP-Related Gene Expressions in mIgM^+^ B Lymphocytes

The detailed methods for sorting mIgM^+^ B lymphocytes from peripheral blood were as previously described (7). The purity of mIgM^+^ B lymphocytes before and after sorting was observed by flow cytometry and also fluorescence microscopy. First, total RNA was extracted from selected mIgM^+^ B lymphocytes using the RNeasy Plus Mini kit (Qiagen, Germany), according to the manufacturer’s instructions. RNA quality was analyzed by Bioanalyzer 2100 (Agilent Technologies, USA). All the primers used in this study are listed in **Table 1**. The specificity of the primers was confirmed by melting curves and the sequencing of amplicons. Real-time quantitative PCR (qRT-PCR) was performed in LightCycler^®^480 II Real Time System (Roche, Switzerland) by SYBR GreenIMaster (Roche, Switzerland). As one of the most conserved genes in vertebrates, our previous studies have shown that 18s rRNA was the least affected by various stimuli in flounder, so 18S rRNA gene was used as the internal reference (7, 31). All data were associated with 18S rRNA gene and analyzed by 2^−ΔΔCt^ method. The differences between the treatment and the control group were analyzed to assess changes in gene expression. Syk, FcγRII, and FcγRIII mRNA levels in mIgM^+^ B lymphocyte were examined by qRT-PCR after incubation with antiserum-opsonized *E. tarda* for 2 h at 22°C, and the healthy flounder serum-opsonized *E. tarda* was used as negative control, as mentioned in RNA isolation. For blocking assay, the Fc fragment of IgM on the *E. tarda* was blocked by the prepared anti-IgM polyclonal antibodies, then the expression level of the three genes in mIgM^+^ B lymphocytes were also detected by qRT-PCR after incubation with antiserum-opsonized *E. tarda* for 2 h at 22°C, while the healthy flounder serum-opsonized *E. tarda* was used as negative control, as mentioned in RNA isolation.

**Table 1:** Primes used for qPCR in this study.

Gene	Primer	Sequence
FcγRII	qFcγRII-F	5’-CCTCATCCACTCTTTGGTTTC-3’
	qFcγRII-R	5’-TGACGAGTAAAGAAGGGAATG-3’
FcγRIII	qFcγRIII-F	5’-TCCTGGGACGCAGACAGACTTAC-3’
	qFcγRIII-R	5’-CGTTGTATTGGAGGTGGCAGATGAG-3’
Syk	qSyk-F	5’-CCGCTGCTACCATTACACCAT-3’
	qSyk-R	5’-CCTCAAACAGCCCCACCTTCG-3’
18S rRNA	q18S rRNA-F	5’-GGTCTGTGATGCCCTTAGATGTC-3’
	q18S rRNA-R	5’-AGTGGGGTTCAGCGGGTTAC-3’

### Detection of the Effect of FcγRII Blocking on mIgM^+^ B Lymphocytes Phagocytosis

Based on the genome sequence of flounder FcγRII (Genbank No. XM_020105965.1), specific primers (F: 5’-CGCGGATCCATGGGCCCAACGTGCAA-3’, BamH I; R: 5’-CCCAAGCTTTCATTGGTCGAACCTCAGAGAC-3’, Hind III) were designed to amplify the target gene. The recombinant FcγRII (rFcγRII) protein and the rabbit anti-rFcγRII polyclonal antibody were prepared and characterized as previously described (32). We treated mIgM^+^ B lymphocytes with prepared anti-rFcγRII polyclonal antibody to block FcγRII on their membrane surface. Briefly, Percoll gradient isolated peripheral blood leukocytes (PBLs) were adjusted to 1.0 × 10^7^ cell/ml with PBS, and incubated with anti-rFcγRII antibodies (20:1, V/V) for 1 h to block the FcγRII. Then, the antiserum-opsonized *E. tarda* were incubated with the blocked leukocytes for 3 h. The unblocked leukocytes were used as control. After washing, mIgM^+^ B lymphocytes were labeled with prepared anti-IgM mAb and Alexa Fluor^®^ 649 goat anti-mouse IgG, and their phagocytosis rates were analyzed by flow cytometry. The FcγRII-blocked mIgM^+^ B lymphocytes were sorted after phagocytosis as previously described, and the ADCP-related and phagolysosome-related genes in them were detected by qRT-PCR.

### Statistical Analysis

Statistical analysis was carried out by Statistical Product and Service Solution (SPSS) software (version 20.0; IBM Corp., USA), and statistical significance was measured by independent-sample t-tests. The results are shown as mean ± S.D., and differences were regarded as significant at P <0.05.

## Results

### Characteristics of Flounder Antiserum and Polyclonal Antibodies Against IgM

**Figure 1:**
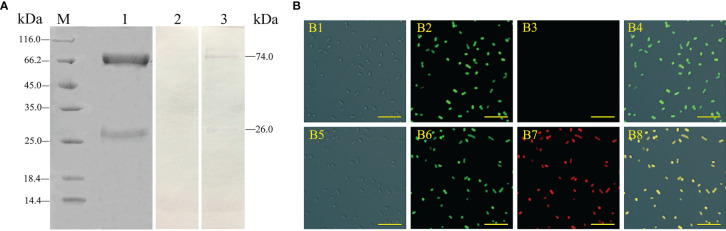
Analysis of the characteristics of rabbit anti-IgM serum and flounder antisera. **(A)** Flounder serum IgM and rabbit anti-IgM sera were analyzed by SDS-PAGE and western blotting. Lane M, molecular mass marker; Lane 1, purified flounder sera IgM; Lane 2, negative control using the serum of unimmunized rabbit; Lane 3, flounder sera IgM was immunostained by anti-IgM polyclonal antibodies. **(B)** Analysis of the binding activity of flounder anti-*E. tarda* serum. B1 and B5: *E*. *tarda* was observed under differential interference microscope. B2: FITC labeled *E*. *tarda*. B6: Immunofluorescence-stained bacteria with flounder antiserum, anti-IgM mAb and Alexa Fluor^®^ 649 goat anti-mouse IgG (B7), and healthy flounder sera were used as control (B3). B4 and B8: Merge images of each fluorescent channel (Bar = 10 μm).

According to SDS-PAGE, the purified IgM had two main bands with molecular weights of approximately 72.0 kDa and 26.0 kDa, corresponding to the predicted heavy and light chains of flounder IgM, respectively, and almost didn’t have other protein bands (**Figure 1A**, lane 1). ELISA results showed that the titers of the flounder antiserum and rabbit anti-IgM polyclonal antibodies were 1/80 and 1/160,000, respectively (data not shown). Western blotting showed that the prepared rabbit anti-IgM polyclonal antibodies were able to specifically recognize the heavy and light chains of flounder sera IgM (**Figure 1A**, lane 3). IFA results showed that the surfaces of all *E. tarda* bacteria showed strong green fluorescence signal, indicating successful FITC labeling. Furthermore, the flounder antiserum could fully adhere to the surface of *E. tarda*, while no specific binding was observed using the healthy flounder sera (**Figure 1B**).

### The Phagocytosis Rates of mIgM^+^ B Lymphocytes Post Opsonization of *E. tarda* and Fc Blocking

**Figure 2:**
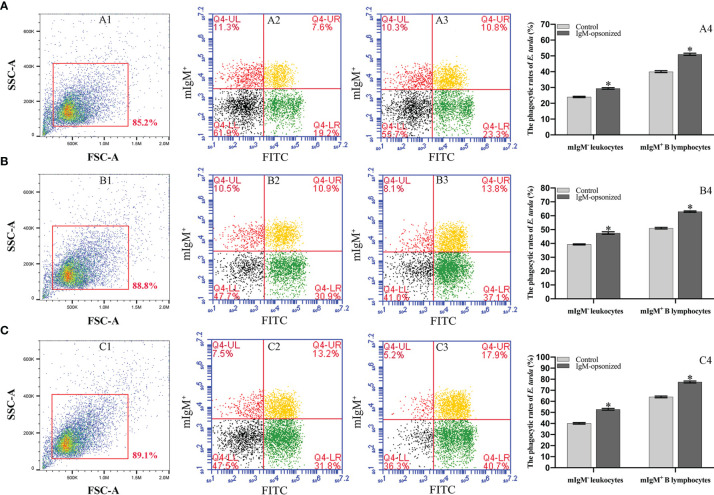
Analysis of phagocytosis rates of flounder mIgM^+^ B lymphocytes after incubation with opsonized *E*. *tarda* using healthy flounder sera and antisera by flow cytometry. **(A–C)** Phagocytosis rates of mIgM^+^ B lymphocytes after incubation with *E*. *tarda* for 1 **(A)**, 3 **(B)** or 5 h **(C)**. 1: FSC/SSC plots of PBLs. Fluorescence scatter plots of PBLs incubated with opsonized *E*. *tarda* using healthy flounder sera (2) and antisera (3). 4: Phagocytosis rates of mIgM^+^ B lymphocytes and mIgM^-^ leukocytes summarized from the fluorescence scatter plots. Error bars indicate the standard deviation of the three biological replicates. The asterisks on the bars represent the statistical difference of the phagocytosis rates compared to the control group (*p <*0.05).

**Figure 3:**
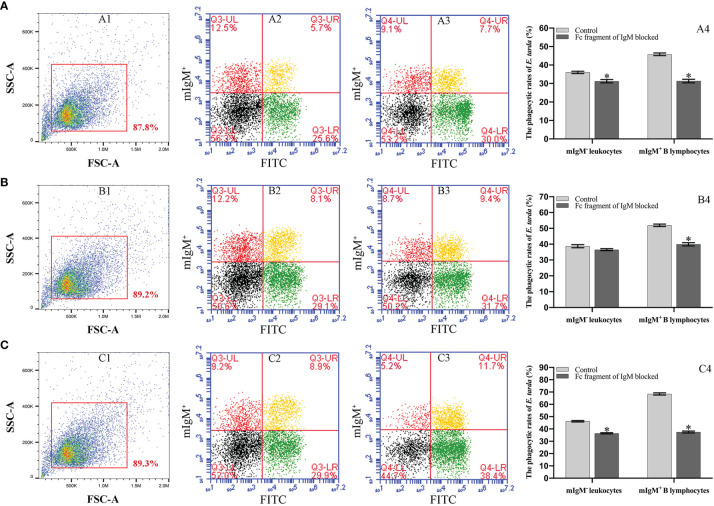
Analysis of phagocytosis rates of flounder mIgM^+^ B lymphocytes after opsonization of *E*. *tarda* and Fc blocking by flow cytometry. **(A–C)** Phagocytosis rates of mIgM^+^ B lymphocytes after incubation with *E*. *tarda* for 1 **(A)**, 2 **(B)** or 3 h **(C)**. 1: FSC/SSC plots of PBLs. Fluorescence scatter plots of PBLs incubated with Fc blocking (2) and unblocked (3) *E*. *tarda*. 4: Phagocytosis rates of mIgM^+^ B lymphocytes and mIgM^-^ leukocytes summarized from the fluorescence scatter plots. Error bars indicate the standard deviation of the three biological replicates. The asterisks on the bars represent the statistical difference of the phagocytosis rates compared to the unblocked group (*p <*0.05).

**Figure 4:**
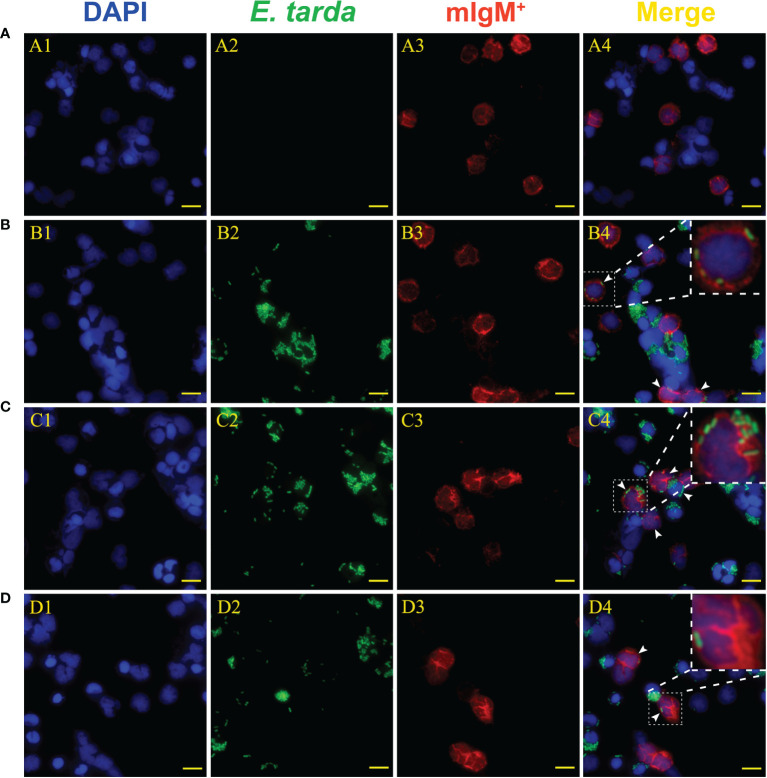
Analysis of phagocytic ability of mIgM^+^ B lymphocytes after incubation with differently treated *E*. *tarda* by fluorescence microscopy. **(A)** Non-phagocytosed negative control. **(B, C)** Opsonization of *E*. *tarda* using healthy flounder sera **(B)** and antiserum **(C)**. **(D)** Fc blocking of the antiserum-opsonized *E*. *tarda*. 1: DAPI staining of cell nuclei. 2: FITC-labeled *E*. *tarda*. 3: Immunofluorescence-stained with anti-IgM mAb and Alexa Fluor^®^ 649 goat anti-mouse IgG. 4: Merge images of each fluorescent channel with magnified images of phagocytosed mIgM^+^ B lymphocytes. Arrows indicate the mIgM^+^ B lymphocytes after phagocytosis (Bar = 10 μm).

Flow cytometry showed the phagocytosis rates of antiserum*-*opsonized *E. tarda* in peripheral blood mIgM^+^ B lymphocytes at 1, 3 or 5 h were 51.1 ± 0.8%, 63.0 ± 0.4%, and 77.5 ± 0.9%, respectively, which were significantly higher than that of control groups with 40.2 ± 0.7%, 50.9 ± 0.6%, and 63.8 ± 0.8%, respectively (**Figure 2**). After the Fc fragment of opsonizing IgM on the surface of *E. tarda* was blocked with prepared antibodies, the phagocytosis rates of opsonized *E. tarda* in peripheral blood mIgM^+^ B lymphocytes at 1, 2 or 3 h were 31.3 ± 1.0%, 39.9 ± 0.8%, and 49.1 ± 0.6%, respectively, which were significantly lower than that of unblocked groups with 45.8 ± 0.7%, 51.9 ± 0.5%, and 69.2 ± 0.6%, respectively (**Figure 3**). Fluorescence microscopy showed that the average number of bacteria within each mIgM^+^ B lymphocyte was greater than 5 after phagocytosis of antiserum-opsonized *E. tarda*, while the control group was less than 5, and the proportion of phagocytosed mIgM^+^ (pha^+^/mIgM^+^) B lymphocytes was also significantly higher than that of the control group. When the Fc fragment of osponizing IgM on the surface of *E. tarda* was blocked, the number of phagocytosed *E. tarda* in mIgM^+^ B lymphocyte was significantly lower than that in the opsonization group, and the proportion of pha^+^/mIgM^+^ B lymphocytes was also obviously decreased compared with that of the opsonization group (**Figure 4**).

### Intracellular ROS Levels in mIgM^+^ B Lymphocytes Post Phagocytosis

**Figure 5:**
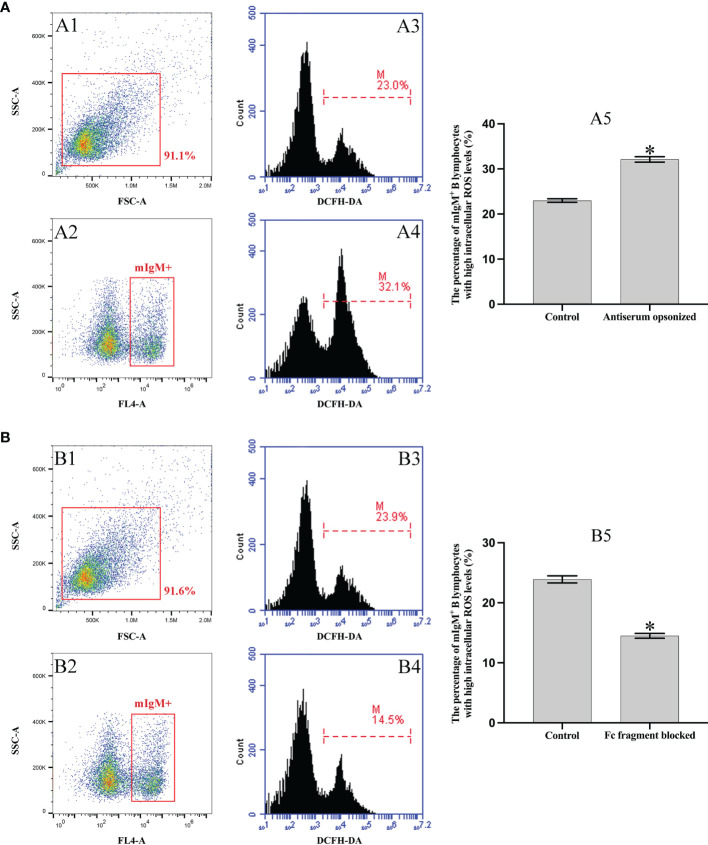
Analysis of intracellular ROS activity of mIgM^+^ B lymphocytes by flow cytometry. **(A)** Incubation with antiserum-opsonized *E. tarda*. **(B)** Incubation with Fc blocked antiserum-opsonized *E. tarda* treated. A1 and B1: FSC/SSC plots of PBLs. A2 and B2: The mIgM^+^ B lymphocytes were gated for analysis. Percentage of mIgM^+^ B lymphocytes with high intracellular ROS levels in healthy flounder sera (A3, B3) and antiserum opsonizing groups (A4, B4). A5 and B5: Percentage of mIgM^+^ B lymphocytes with high intracellular ROS levels summarized from the fluorescence histogram. Error bars indicate the standard deviation of the three biological replicates. The asterisks on the bars represent the statistical difference of the intracellular ROS levels compared to the control group (*p < *0.05).

Flow cytometry showed that 32.1 ± 0.7% of mIgM^+^ B lymphocytes had higher intracellular ROS levels after phagocytosis of antiserum-opsonized *E. tarda*, which were significantly higher than that of control group with 23.0 ± 0.5%. In addition, when the Fc fragment of opsonizing IgM on the surface of *E. tarda* was blocked, 14.5 ± 0.4% of mIgM^+^ B lymphocytes had high intracellular ROS levels after phagocytosing the treated *E. tarda*, which were significantly lower than that of opsonization group with 23.9 ± 0.6% (**Figure 5**).

### Expression Profiles of ADCP Related Genes in mIgM^+^ B Lymphocytes Post Phagocytosis

**Figure 6:**
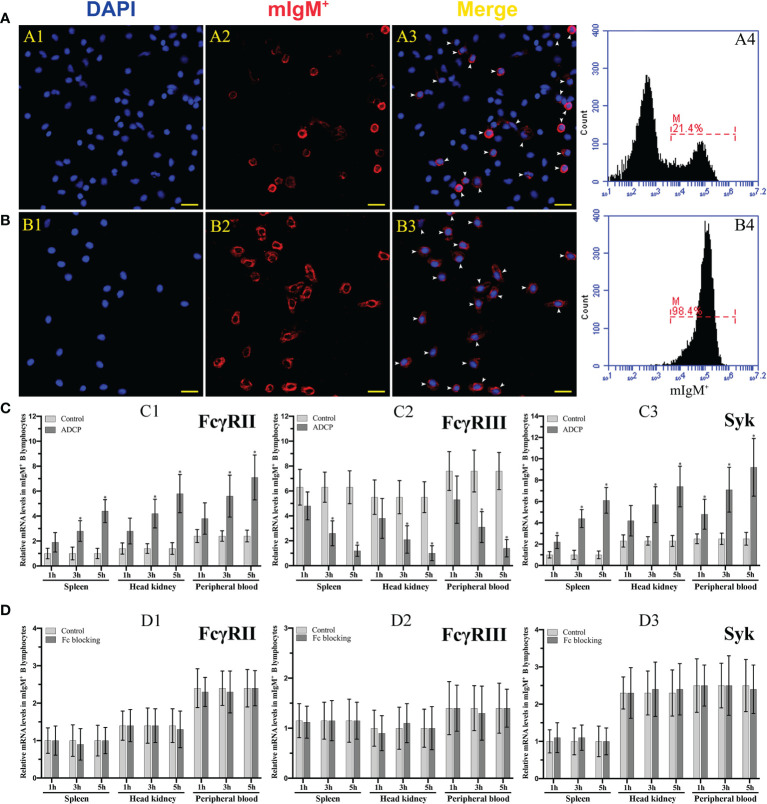
Analysis of the purity of sorted mIgM^+^ B lymphocytes and expression levels of ADCP-related genes after their phagocytosis. **(A, B)** Analysis of the percentage of mIgM^+^ B lymphocytes in unsorted **(A)** and sorted **(B)** cells by IFA and flow cytometry. A1 and B1: DAPI staining of cell nuclei. A2 and B2: Immunofluorescence-stained with anti-IgM mAb and Alexa Fluor^®^ 649 goat anti-mouse IgG. A3 and B3: Merge images of each fluorescent channel and arrows indicate the mIgM^+^ B lymphocytes (Bar = 20 μm). Fluorescence histogram showed the percentage of mIgM^+^ B lymphocytes (scale of M) in unsorted leukocytes (A4) and sorted mIgM^+^ B lymphocytes (B4). **(C, D)** The qRT-PCR analysis of the expression levels of ADCP-related genes in mIgM^+^ B lymphocytes post incubation with antiserum-opsonized **(C)** and Fc blocked *E. tarda* **(D)**, respectively. C1 and D1: FcγRII. C2 and D2: FcγRIII. C3 and D3: Syk. Error bars indicate the standard deviation of the three biological replicates. The asterisks on the bars represent the statistical difference of genes expression compared to the control group (*p <* 0.05).

The purity of the magnetic bead sorted and unsorted mIgM+ B lymphocytes form peripheral blood leucocytes was detected by flow cytometry to be 98.4% and 21.4%, respectively, which was fairly consistent with the observation by fluorescence microscopy, and could be used for subsequent experiment (**Figures 6A, B**). The qRT-PCR results showed that the expression levels of FcγRII and Syk in mIgM^+^ B lymphocytes were progressively upregulated with the prolongation of phagocytosis time in the opsonization group, whereas FcγRIII was progressively downregulated (**Figure 6C**). When the Fc fragment of opsonizing IgM on the surface of *E. tarda* was blocked, the expression levels of FcγRII, FcγRIII, and Syk in mIgM^+^ B lymphocytes did not vary with phagocytosis time (**Figure 6D**).

### Recombinant FcγRII (rFcγRII) Purification and Characteristics of Prepared Anti-rFcγRII Antibodies

**Figure 7:**
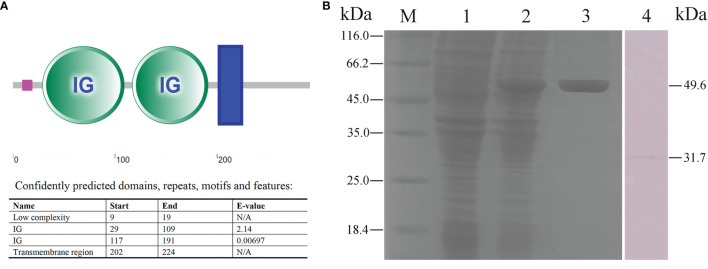
Analysis of FcγRII recombinant expression and characteristics of anti-rFcγRII antibodies. **(A)** Analysis of flounder FcγRII domain. The pink line, green circle, and blue box represent the low complexity domain, the IG domains, and the transmembrane region, respectively. **(B)** SDS-PAGE and western blotting analysis of rFcγRII protein and prepared anti-rFcγRII antibodies. Lane M, molecular mass marker; Lane 1, transformed *E. coli* without IPTG induction; Lane 2, transformed *E. coli* induced with IPTG; Lane 3, purified rFcγRII protein; Lane 4, mIgM^+^ B lymphocytes lysate incubated with prepared anti-rFcγRII antibody.

Flounder FcγRII protein consisted of a low complexity (residues 9–19), two IG domains (residues 29–109, 117–191), and a transmembrane region (residues 202–224) (**Figure 7A**). The FcγRII was expressed in *E. coli* (DE3) using the pET-32a plasmid system and SDS-PAGE analysis showed a distinct 49.6 kDa band after isopropyl β-D-1-thiogalactoside (IPTG) induction (**Figure 7B**, lane 2). After performing Ni^2+^ affinity chromatography purification and stepwise dialysis refolding methods, high purity rFcγRII protein was obtained (**Figure 7B**, lane 3). Moreover, western blotting showed that the prepared rabbit anti-rFcγRII antibodies could recognize a 31.7 kDa molecule of PBLs, which was consistent with the theoretical molecular weight of flounder FcγRII (**Figure 7B**, lane 4).

### The Phagocytic Rates and Phagocytosis-Related Genes Expression of mIgM^+^ B Lymphocytes Post FcγRII Blocking

**Figure 8:**
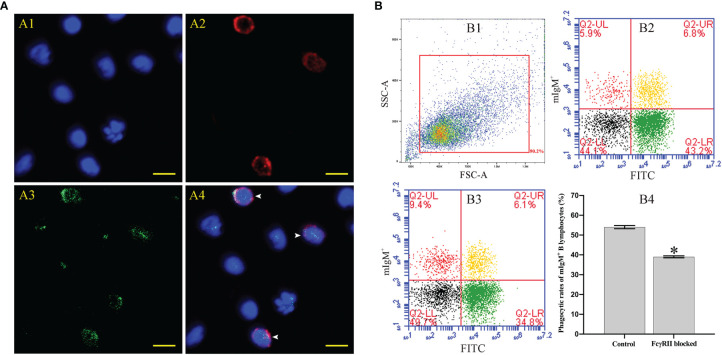
The distribution characteristics of FcγRII and its effect on mIgM^+^ B lymphocytes ADCP. **(A)** IFA analysis of distribution characteristics of FcγRII in PBLs. A1: DAPI staining of cell nuclei. A2: Immunofluorescence-stained leukocytes with anti-IgM mAb and Alexa Fluor^®^ 649 goat anti-mouse IgG. A3: Immunofluorescence-stained leukocytes with rabbit anti-rFcγRII antibodies and Alexa Fluor^®^ 488 goat anti-rabbit IgG. A4: Merge images of each fluorescent channel and arrows indicate the FcγRII^+^/mIgM^+^ B lymphocytes (Bar = 10 μm). **(B)** Flow cytometry analysis of phagocytosis rates of FcγRII-blocked mIgM^+^ B lymphocytes after incubation with antiserum-opsonized *E. tarda*. B1: FSC/SSC plots of PBLs. B2: Co-incubation with FcγRII-blocked PBLs and antiserum-opsonized *E. tarda*. B3: Co-incubation with PBLs and antiserum-opsonized *E. tarda*. B4: Phagocytosis rates of mIgM^+^ B lymphocytes summarized from the fluorescence scatter plots. Error bars indicate the standard deviation of the three biological replicates. The asterisks on the bars represent the statistical difference of the phagocytosis rates compared to the control group (*p <* 0.05).

**Figure 9:**
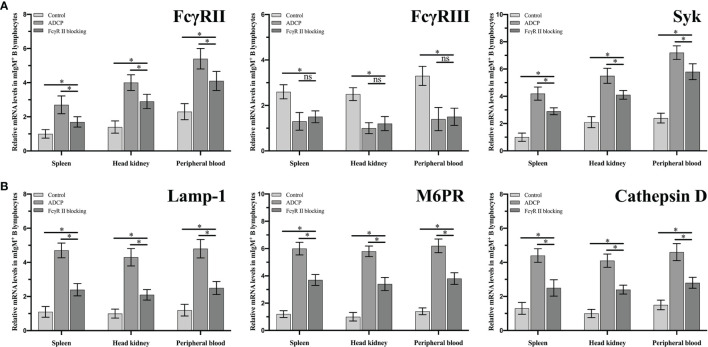
The expression levels of ADCP-related **(A)** and phagolysosome-related **(B)** genes in FcgRII blocked and unblocked mIgM+ B lymphocytes after phagocytosis. Error bars indicate the standard deviation of the three biological replicates. ns, no significant difference. The asterisks on the bars represent the statistical difference of genes expression compared to the control group (p < 0.05).

Fluorescence microscopy showed that flounder FcγRII was expressed on mIgM^+^ B lymphocytes membranes and also on other types of mIgM^-^ leukocytes membranes (**Figure 8A**). When FcγRII of mIgM^+^ B lymphocytes was blocked, the phagocytosis rate of antiserum-opsonized *E. tarda* was 39.0 ± 0.5%, which was significantly lower than that of unblocked group with 54.0 ± 0.8% (**Figure 8B**). After FcγRII blocking, the qRT-PCR results showed that compared with the unblocked group, the expression levels of FcγRII, Syk, and phagolysosome-related genes were found to be significantly downregulated, whereas FcγRIII showed no significant differences in mIgM^+^ B lymphocytes post phagocytosis (**Figure 9**).

## Discussion

FcR is an important modulator receptor that is widely expressed on all types of leukocytes, which could mediate phagocytosis (33, 34). B cells are an important class of immune cells with the ability to secrete antibodies, present antigens, and phagocytosis (35), but there is no study about the function of FcR in B cell phagocytosis. In the present study, complement in flounder antisera was inactivated by water bath and then used for the opsonization of *E. tarda*. This was in order to avoid the complement receptors which might interfere with the function performed by the FcR during the experiment (36). We found that the phagocytosis rate of antiserum-opsonized *E. tarda* by flounder mIgM^+^ B lymphocytes was significantly higher than the healthy flounder sera opsonizing group. Similarly, the phagocytosis rate of IgM-opsonized *Aeromonas hydrophila* by rainbow trout IgM^+^ cells was significantly higher than that of the unopsonized group (4). Moreover, after the Fc fragment of IgM on the opsonized *E. tarda* surface was blocked by the prepared antibodies, we found that the phagocytosis rates and the number of ingested bacteria in mIgM^+^ B lymphocytes were significantly lower than that of the unblocked groups. These results demonstrate that the FcR plays a pivotal role in mediating the phagocytosis of flounder mIgM^+^ B lymphocytes.

Among all types of FcR, the FcγRII plays an important role in the phagocytosis of phagocytes in mammals (37, 38). We found that the FcγRII was expressed on the membrane of mIgM^+^ B cells in flounder, which was consistent with the finding on human B cells (39). When the FcγRII of mIgM^+^ B lymphocytes was blocked with specific antibodies, their phagocytosis rates of antiserum-opsonized *E. tarda* were significantly decreased, which indicated that the protein structural domain of flounder FcγRII was similar to that of mammalian (40), thus they may have the same biological functions. Previous studies demonstrated that FcγRII could enhance phagocytosis and production of phagolysosome in neutrophils and Chinese hamster ovary (CHO) cells, respectively (41, 42). We also found that the signaling pathways of ADCP and phagolysosome formation in flounder mIgM^+^ B lymphocytes were both inhibited after FcγRII was blocked. In mammals, FcγRIII consists of two families, FcγRIIIa and FcγRIIIb (43). FcγRIIIa is a transmembrane protein and mainly distributed on monocytes, macrophages, and NK cells. FcγRIIIb has no transmembrane domain and is mainly distributed on neutrophils and basophils (44). However, up to now, no subfamily of FcγRIII gene was found in teleost (45), and the same was found for the FcγRIII in flounder based on the genome sequence. Interestingly, we found that FcγRIII was expressed in flounder mIgM^+^ B lymphocytes, which would be shed from the surface of mIgM^+^ B lymphocytes post phagocytosis regardless of whether FcγRII was blocked or not. The possible reason is that flounder FcγRIII, like mammalian FcγIIIb, lacks a transmembrane structural domain and is easily shed from the cell surface post stimulation, then produces an inflammatory response, leading to the downregulation of its expression in mIgM^+^ B lymphocytes (46, 47). These results indicate that FcγRII rather than FcγRIII plays an important role in ADCP process of flounder mIgM^+^ B lymphocytes.

The protein tyrosine kinase Syk plays a central role in FcγR-mediated phagocytosis in the adaptive immune system (48). We found that the expressions of Syk and FcγRII in flounder mIgM^+^ B lymphocytes were significantly upregulated after FcR-mediated phagocytosis. Similarly, maternal antibodies opsonized-microglia could enhance phagocytic abilities and upregulating the expression of Syk and FcγRs in phagocytes (49). FcγRIII expression in mIgM^+^ B lymphocytes was also found to be gradually downregulating with the prolonged incubation with antisera-opsonized *E. tarda*. This suggests that FcγRIII is shed from the mIgM^+^ B lymphocytes membrane surface upon activation by the Fc fragment. The same phenomenon was also found in NK cells post stimulation by IgG-opsonized platelets (50). When the Fc fragment of IgM on the opsonized *E. tarda* surface was blocked by the prepared antibodies, the expression levels of FcγRII, FcγRIII and Syk in mIgM^+^ B lymphocytes were invariable with prolongation of incubation time. This suggests that the resting FcR cannot induce activation of ADCP-related genes in mIgM^+^ B lymphocytes (51, 52). In addition, a significantly higher expression level of FcγRII in peripheral blood mIgM^+^ B lymphocytes than that in spleen and head kidney was detected, while the expression level of FcγRIII showed no significant difference. The expression level of Syk was significantly higher in peripheral blood and head kidney mIgM^+^ B lymphocytes than that in spleen. We speculate that the phagocytic capacity of peripheral blood mIgM^+^ B lymphocytes is much stronger than that in spleen and head kidney of flounder. Coincidentally, the phagocytosis rate of *Lactococcus lactis* by peripheral blood mIgM^+^ B lymphocytes was significantly higher than that of spleen and head kidney in *L. japonicus* (5). Taken together, these results indicate that flounder FcγRII mediates ADCP *via* Syk, whereas the FcγRIII has rarely effects on ADCP in flounder mIgM^+^ B lymphocytes.

The respiratory burst was an unspecific and defensive mechanism towards the pathogenic microorganism of phagocytes and it also participated in innate immunity through the production of intracellular ROS mediated by the multicomponent enzyme NADPH oxidase (53, 54). Previous investigation has made clear that the intracellular ROS levels of mIgM^+^ B lymphocytes were significantly higher after phagocytosis of bacteria (7). We also found that the intracellular ROS levels of mIgM^+^ B lymphocytes were significantly higher after phagocytosis of antiserum-opsonized *E. tarda* in comparison with the control group. Furthermore, when the Fc fragment of IgM on the surface of opsonized *E. tarda* was blocked by the prepared antibodies, the intracellular ROS levels in mIgM^+^ B lymphocytes after phagocytosis were significantly decreased. These results demonstrate that FcR-mediated phagocytosis significantly enhances the innate immune response of mIgM^+^ B lymphocytes.

In conclusion, flounder FcR could enhance the abilities of phagocytosis, intracellular bactericidal effect, and induce the expression of ADCP-related genes in mIgM^+^ B lymphocytes. Furthermore, we identified FcγRII as a crucial component in the FcR family that exerted influence in mIgM^+^ B lymphocytes ADCP. This study is conducive to our understanding of FcR-mediated phagocytosis in teleost mIgM^+^ B lymphocytes as well as providing an insight into the functions of B cell in innate immunity.

## Data Availability Statement

The datasets presented in this study can be found in online repositories. The names of the repository/repositories and accession number(s) can be found in the article/supplementary material.

## Ethics Statement

The animal study was reviewed and approved by the Committee of the Ethics on Animal Care and Experiments at the Ocean University of China.

## Author Contributions

XT and YH made efforts to the idea and design of this experiment, carried out plenty of experiments and statistical analysis, drafted, and modified this paper. JX, XS, and HC took part in the layout of the study, and assisted to analyze experiments and information. WZ devised the study, compiled the paper, and offered experimental apparatus and space. All authors contributed to the article and approved the submitted version.

## Funding

This study was supported by the National Natural Science Foundation of China (31730101, 31872590, 31672685, 31672684, and 3142295), the Natural Science Foundation of Shandong Province (ZR2019MC029), the Fundamental Research Funds for the Central Universities (201822015), and the Taishan Scholar Program of Shandong Province.

## Conflict of Interest

The authors declare that the research was conducted in the absence of any commercial or financial relationships that could be construed as a potential conflict of interest.

## Publisher’s Note

All claims expressed in this article are solely those of the authors and do not necessarily represent those of their affiliated organizations, or those of the publisher, the editors and the reviewers. Any product that may be evaluated in this article, or claim that may be made by its manufacturer, is not guaranteed or endorsed by the publisher.
